# The Southwestern Medical Record

**Published:** 1896-02

**Authors:** 


THE SOUTHWESTERN MEDICAL RECORD.—The JOURNAL welcomes to its exchanges the *Southwestern Medical Record*, the new Texas journal, lately started at Houston. We have received Vol. 1, No. 1. While the mechanical part is, as might be expected in the beginning, a little crude, and susceptible of improvement, the contents show spirit, enterprise and an aptness at journalism hardly expected of tyros. It gives promise of good fruits; and there are none who will rejoice more at its success, nor be more ready to say "I told you so," than the Red Back; for if professional attainments, high social and professional character and extensive reading, coupled with ambition to succeed, and pride in success, can be counted on—the *Southwestern Medical Record* will succeed.

The Red Back holds out its strong right hand, ready to give its struggling young brother a lift; and we meet him more than half way with a warm welcome.

